# Integrating AI Into Governmental Public Health Decision Making: Challenges, Considerations, and a Path Forward

**DOI:** 10.2196/88470

**Published:** 2026-04-27

**Authors:** Elizabeth Campbell, Oluremilekun Oyefolu, Sarah Gillani, Hannah Goodtree, Alison Kelly, Caitlin Rivers, Crystal Watson

**Affiliations:** 1 Johns Hopkins Center for Outbreak Response Innovation, 700 East Pratt Street, Suite 900, Baltimore, MD, 21202, United States, 1 6097524672

**Keywords:** public health, elected officials, governance, AI, artificial intelligence, decision making, public health emergency response

## Abstract

Public health emergencies such as pandemics, natural disasters, and epidemics may require rapid, high-stakes decisions often made by elected officials with limited public health training. Artificial intelligence (AI) holds significant promise to enhance the quality, transparency, and timeliness of governmental decision-making during such crises. This paper examines the potential of AI as a decision-support tool for elected officials while identifying key technical, logistical, ethical, and policy challenges. Technical considerations include model accuracy, data representativeness, and privacy protection, while ethical imperatives center on fairness, transparency, and accountability to prevent amplification of existing health disparities. The paper further explores workforce development needs, emphasizing AI literacy and cross-sector collaboration to enable informed use of AI insights. This viewpoint presents a novel AI Decision Support Lifecycle framework specifically designed for governmental public health emergency response, mapping six phases from problem definition through post-emergency evaluation. We provide stakeholder-specific recommendations for model developers, health agencies, and elected officials, and illustrate practical application through a detailed case example and use cases. Drawing on empirical evidence regarding digital health technologies and AI governance, we emphasize that technology deployment alone is insufficient. Successful implementation requires complementary investments in organizational capacity, data infrastructure, workforce training, community engagement, and continuous evaluation. AI integration also requires robust governance frameworks, continuous model evaluation, and alignment with existing crisis management structures. Policy recommendations highlight the importance of ethical AI frameworks, risk assessments, and public engagement to foster trust. Ultimately, AI can strengthen public health decision-making if developed and implemented responsibly within transparent and equitable systems.

## Introduction

Public health emergencies, such as the COVID-19 pandemic, the 2014‐2016 West Africa Ebola epidemic, and natural disasters such as floods or hurricanes, pose serious and pressing individual and population health threats. Depending on the size and scope of these emergencies, they can require urgent and harmonized responses from governmental decision makers and public health systems at the local/tribal, state/territorial, and even national levels [1,2]. Sometimes, when an emergency is of significant size and scope, or is highly visible or consequential, decisions that are typically made by public health or emergency management experts shift to, or at least involve, elected officials, such as state governors [3]. This shift might be made because elected officials are politically responsible for the outcome of a response, or the shift might happen because only elected officials can exercise the legal powers necessary to respond. For example, in all states and territories, governors can issue emergency declarations to enact measures such as accessing and reallocating funds or suspending laws and regulations that may impede response efforts [4-6].

Regardless of the inciting event, when elected officials or other leaders are thrust into a public health decision-making role, often without formal public health or crisis response training, they may need additional support to ensure that decisions are well-considered. While public health and emergency management experts can, and should, play a significant supporting role in decision-making, leaders without good decision-making processes in place can easily fall victim to so-called decision-derailers–common biases, traps, and heuristics that can make decisions less-than sound [7].

## Challenges to Decision Making During Public Health Emergencies

A hallmark of public health emergencies is an initial severe information deficit, while many competing voices clamor for urgent action. There is also inevitably a tug-of-war between health and safety priorities and other societal imperatives such as economic stability. Further, the broad categories of “health,” “economic,” and “societal” impacts must themselves be unpacked; choices about how to respond, what measures and recommendations should be put in place, must consider and involve diverse populations who are affected in very different ways and to different degrees.

So, what can leaders do to ensure that their decision-making process has systematically identified and considered a full range of actions that could effectively address fundamental goals; carefully considered and balanced all the values at stake; incorporated the information available while hedging against the remaining uncertainties; and has built public support through its transparency and thoroughness?

## Operationalizing Effective Decision Making During Public Health Emergencies

First, policymakers should clearly define, justify, and articulate the strategic goals that they aim to achieve and then share this information with the public. Next, it is critical to identify what principles leaders want to uphold in an emergency response. Protecting the health of the public is a general principle that most leaders identify with. Are there other important principles that should not be forgotten, like ensuring the consequences of interventions are fair across populations? As decisions are not made in a vacuum, the voices of diverse experts help to consider a variety of angles to a problem.

Once the goals, principles, and structure for decision-making are in place, an assessment process for identifying and evaluating options for action is needed. Evaluating the possible downstream impacts of options (both positive and negative), comparing options against one another, and assessing the logistical feasibility of the range of options. Finally, in addition to a continual updating as new evidence and information emerges, well-documented communication practices highlight the importance of telling the public that information may change and foreshadowing how new information can change plans.

In a crisis, these structures and processes are very difficult to implement, especially under time pressure and without prior planning. Developing plans ahead of a crisis is optimal, but limited time and resources make this rare, and leaders will still face an information deficit difficult to overcome with legacy approaches to data gathering.

## AI for Public Health Decision-Making

Artificial intelligence holds considerable promise to help decision-makers gather data to inform decisions and implement decision-making processes to systematically consider options and undertake tradeoff analyses. Ultimately, AI has the potential to greatly enhance the quality of public health decision-making in these emergencies. However, while the potential for decision support is significant, numerous challenges persist in developing and operationalizing AI for public health crisis decision making. While digital health technologies and information and communication technology (ICT) infrastructure have expanded rapidly, empirical evidence regarding their impact on population health outcomes remains mixed [8-12]. Studies show that ICT adoption does not automatically translate into measurable health improvements, particularly when institutional capacity, governance quality, workforce skills, and implementation fidelity are insufficient. Previous digital health initiatives often showed weak or context-dependent effects due to inadequate organizational readiness, data quality issues, and a lack of complementary investments in regulatory frameworks and human capital.

The following viewpoint considers technical, logistical, and ethical challenges in developing AI tools to support elected officials in public health decision making and describes important considerations when addressing AI applications to optimize system use in the public health domain.

### Aim and Scope

This viewpoint article aims to: (1) examine the potential role of AI as a decision-support tool for elected officials during public health emergencies and (2) identify key technical, ethical, and governance challenges that must be addressed.

This viewpoint focuses specifically on US federal, state, and local governmental public health decision-making contexts. While many principles may apply internationally, our discussion of policy frameworks, legal requirements, and organizational structures reflects the US governance system. Jurisdictions with different regulatory environments, data infrastructure capacity, or political structures may face distinct challenges requiring adaptation of these recommendations.

### Target Audience

This viewpoint is written for three primary audiences: (1) state and local public health leaders and emergency management officials who must integrate AI tools into decision-making workflows; (2) AI developers and data scientists working with governmental agencies on public health applications; and (3) elected officials and policymakers who oversee public health emergency response and must understand both the potential benefits and risks of AI-supported decision making.

### Methodological Approach

This is a viewpoint article that synthesizes insights from the emerging interdisciplinary literature on AI in public health, governmental AI governance, and algorithmic fairness. Literature was identified through targeted searches of key databases (PubMed, Web of Science, OAISTER) and policy sources (federal and state government websites, Office of Management and Budget [OMB] guidance, state legislative databases), focusing on AI applications in governmental public health decision-making, with particular attention to recent publications (2020‐2025) addressing COVID-19 response and other public health emergencies.

While existing AI governance frameworks (eg, the National Institute of Standards and Technology (NIST) AI Risk Management Framework, Organisation for Economic Co-operation and Development (OECD) AI Principles) offer domain-general guidance, and crisis management models address emergency decision structures, neither adequately addresses the intersection of AI-specific technical challenges and the distinctive governance realities of public health emergency response [1,13]. This includes the shift of decision authority to elected officials with limited technical training, political accountability pressures, and the need for real-time model adaptation. The AI Decision Support Lifecycle framework presented here is designed to fill this gap by providing phase-specific, stakeholder-differentiated guidance tailored to this context.

## Technical Challenges in Model Development

Machine learning (ML) models that support public health decision-making are subject to technical challenges in their development pipeline prior to being scaled for public health response efforts. Underfitting and overfitting are two of the most common challenges in the model development process, which limit a model’s utility. Models are sensitive to quality and representativeness in the data used to train them.

Overfitting occurs when a model becomes too specialized in its training data and fails to perform well on new, unseen data. Overfitting limits a model’s generalizability on test or real-world data and leads to high variance in model performance based on changes to input data. For example, a forecasting model trained on historical influenza data that captures every nuance of past flu seasons may perform poorly during a novel pandemic like COVID-19, where transmission dynamics, public behavior, and health care utilization patterns differ substantially from historical precedent. Overfitting may be addressed by collecting more data to create a more robust training set, reducing the model parameters or undertaking feature selection, and using cross-validation. Underfitting occurs when a model is too simplistic and fails to capture underlying patterns in data. This leads to missed patterns and inaccurate predictions, even for training data. In emergency contexts, this might manifest as a basic model that predicts hospital admissions using only a single variable (eg, previous d’s case count) while ignoring critical factors like vaccination rates, demographic composition, or seasonal patterns. Such models provide unreliable guidance and may lead decision-makers to underestimate resource needs. Increasing the number of model parameters or features and reducing regularization may help to reduce underfitting.

## Ethical Considerations for AI in Public Health

### Bias and Fairness in AI Algorithms for Public Health Decision Making

Ensuring algorithmic fairness and minimizing potential algorithmic biases is a key goal for successfully implementing AI into public health decision-making processes. Data used for developing AI models for public health practice may come from a variety of sources, including electronic health records, governmental data on demographic, socioeconomic, and environmental indicators, social media, and genomic data. These models are trained on vast amounts of data, which may inadvertently contain bias. Without actively and adequately addressing these biases, AI models for public health decision making may amplify and perpetuate existing health disparities.

Bias in public health AI models may come from a variety of sources. If training data is not representative of all subpopulations (eg, age, race/ethnicity, gender), this leads to sampling bias and may cause inaccurate model predictions for members of underrepresented groups [14-16]. Furthermore, AI models trained on past health care data may learn historical biases embedded in these data [17]. For example, Obermeyer et al identified bias in model performance against Black patients for a commercial algorithm used to predict population health risk. The model used health care costs as a proxy for health (ie, lower health care costs equating to better health). However, in the US health care system, less money is spent caring for Black patients compared to White patients, and Black patients are less likely to seek out and obtain medical care [18]. Health care data is also subject to measurement bias, which occurs when there are systemic errors in how health outcomes are measured and recorded (eg, misdiagnosis or underreporting) [19]. Finally, bias may be introduced algorithmically (eg, feature selection bias) or in model design processes [20-22]. AI algorithms also often work as black boxes, which further complicates their deployment in public health decision making efforts since it is difficult for both government officials and constituents to understand how a decision was reached. This lack of transparency and explainability can reduce trust in AI-driven policies, cause concerns about accountability, and create complex ethical and legal challenges should AI-driven recommendations lead to harm [23]. Should AI solutions produce incorrect, harmful, or misleading recommendations, this leads to public mistrust and decreased likelihood of buy-in for public health measures that require community-wide support (eg, social distancing).

### Equity in Practice

To mitigate some of these potential risks, organizations responsible for developing and scaling public health AI models and governmental decision makers may undertake several actions. First, model designers should carefully curate large, representative datasets that reflect the diverse populations that public health decisions will impact. When models are developed, it is also critical that designers assess models for performance disparities, identify fairness metrics, and undertake careful documentation of model development processes to ensure transparency and accountability. Furthermore, it is essential that decision makers receive thorough training so that they are able to understand and interpret AI system recommendations to promote trust, accountability, and transparency. To operationalize equity in public health AI systems, organizations must implement concrete safeguards throughout the AI lifecycle, including: establishing compensated community advisory boards with decision-making authority from historically marginalized populations; engaging diverse stakeholders through participatory design processes to identify potential harms and validate outputs; conducting formal equity impact assessments before and during deployment with transparent public reporting; evaluating and reporting model performance metrics separately for demographic and geographic subgroups (race/ethnicity, age, gender, socioeconomic status, disability, language, geography) with minimum performance thresholds for all groups rather than population averages alone; developing culturally appropriate communication strategies that provide plain language, multilingual explanations of AI use through trusted community channels with clear feedback and recourse mechanisms; and implementing continuous bias auditing through both internal reviews and independent third-party assessments with public documentation of findings and remediation steps. Finally, model development teams and government organizations should work together to regularly monitor and evaluate AI models to identify and correct potential biases, update and retrain models as new data becomes available, and ensure that models continue to adhere to existing legal and ethical standards [24].

### Privacy and Data Security

AI and ML programs and utilization can both enhance data security or generate issues related to privacy, confidentiality breaches, and data security [25]. There are opportunities for both malicious and accidental incidents that can result in confidence reduction, misclassification of data, or misclassification of outputs, all with the potential for severe downstream effects [26]. AI and ML models require the collection, storage, and analysis of vast quantities of data, including health data, personal identifying information, and confidential information. Security risks are a huge concern for these models and should be a priority when developing and using AI or ML tools. Opportunities for data breaches or loss of security could occur at an individual level, including someone with authorized access using data maliciously, it could occur accidentally through non-secure networks, or it could occur through organized and intentional breaches into private systems [27].

The vast number of systems that may interact with an individual’s personal data also increases the risk of privacy concerns, since more systems translates to more opportunities for risk [28]. It is essential that developers, researchers, and institutions develop, test, and continue to improve and invest in safeguards for data access, use, and control. These safeguards can include privacy risk mitigation strategies and privacy-enhancing technologies [29], including de-identifying data, safeguarding physical access to systems, and strong encryption and protection of cloud-based systems [28]. There are also ethical considerations for inadequate data privacy protections, including data breaches or unauthorized access. Individuals who have personal or confidential data used in these models should have provided fully informed consent [30], be updated in the event of a security compromise, and have the option to withdraw their data from the model. There are ongoing discussions on data ownership and profit from individual data as well [31]. Finally, regulatory bodies should exist that can enforce privacy protections and provide recourse when entities fail to protect sensitive data or violate consent for data use [30].

## Implementation: Organizational Readiness

### Integrating AI Into Education and Government Workforce Development

Successful AI deployment in the public sector hinges not only on technological capabilities but equally on organizational readiness and workforce expertise. Evidence from previous digital health initiatives demonstrates that technology alone is insufficient; effective implementation requires complementary investments in organizational capacity, data infrastructure, and workforce skills [8-12].

The G7 Toolkit for Artificial Intelligence in the Public Sector identifies workforce digital skills as a foundational requirement for effective and responsible AI deployment in government contexts. A multi-layered approach is essential to building this capacity. Foundational AI literacy programs can equip public officials with an understanding of both AI’s potential and its inherent limitations. Expert workshops utilizing scenario-based exercises represent another valuable resource that could help decision-makers navigate the complex trade-offs between privacy protection, equity considerations, and operational efficiency. Historical analysis also offers great learning opportunities. By examining case studies demonstrating predictive disease surveillance and resource allocation during past emergencies, officials can visualize concrete applications of AI and develop strategies to manage implementation challenges as they arise.

### Considerations for Implementation Into Workflows, Policy Formulation, Program Planning, and Resource Allocation

A successful deployment of artificial intelligence during public health emergencies extends well beyond technological considerations, requiring robust institutional frameworks and operational adaptations. Historically, expert consensus functioned as a governance mechanism through which policy alternatives were prioritized. The absence of a standardized governance framework for AI implementation may create significant hesitation among elected officials to support AI-derived recommendations, particularly in situations when actions are under public scrutiny [32].

AI integration into public health decision-making would also require a seamless alignment with existing processes and adaptive governance structures. The fragmentation of data across multiple stakeholders and information systems could result in delayed or incompatible information transfer, compromising model accuracy when rapid analytical insights are most critical [33]. At the operational level, AI outputs must be integrated directly into existing crisis-management dashboards and consoles so that public health officials can obtain timely and actionable insights without redundant data entry or additional interfaces [34].

The implementation of AI tools during a public health emergency involves significant resource considerations beyond software licensing and hardware acquisition. Investments in data management infrastructure, cybersecurity protections, and dedicated personnel are also essential. Additionally, ongoing model retraining and impact assessments are required to maintain relevance as conditions evolve [35]. The resource allocation challenge is further complicated in emergency settings, where bandwidth and computational resources face competition from numerous concurrent priorities such as other critical emergency response functions.

Artificial intelligence systems can function as decision support mechanisms that enhance rather than supplant the judgment of elected officials and their technical advisors when designed with appropriate architectural safeguards. The safeguard features could include human validation functions that allow flagging specific output for expert review at key decision points [36], an iterative feedback loop function to allow officials to log disagreements with model outputs and feed these back into model retraining [33], an ensemble function that can combine model predictions with expert systems to ensure known constraints are reinforced alongside data-driven insights [37].

**Case Study:** To illustrate how these implementation considerations manifest in practice, we present a detailed case example:

### Hospital Surge Prediction During Influenza Season

A state health department seeks to deploy an AI forecasting model to predict hospital bed needs 2‐4 weeks ahead during the influenza season, allowing proactive resource mobilization.

*Problem Definition*: The health department, in consultation with hospital associations and emergency management, identifies specific decision needs: estimating intensive care unit (ICU) bed demand, identifying which geographic regions will face capacity constraints, and determining timing for activating surge capacity plans.

*Model Development*: Data scientists develop an ensemble forecasting model using historical hospitalization data, influenza surveillance data, and demographic information. The model undergoes rigorous validation using holdout data from previous flu seasons, with performance assessed overall and for relevant subgroups (age categories, urban vs rural regions).

*Integration*: The model is integrated into the state’s existing emergency operations center dashboard, providing daily updated forecasts with clear uncertainty ranges. Training is provided to epidemiologists and emergency coordinators on interpreting forecasts and understanding uncertainty.

*Deployment*: During flu season, forecasts inform daily situation reports to the governor’s office. When the model predicts ICU capacity concerns in the jurisdiction’s northern region, officials activate surge plans, coordinate patient transfers, and deploy mobile medical units. Importantly, epidemiologists review each forecast before distribution, flagging any predictions that seem inconsistent with other data sources.

*Failure Mode*: Mid-season, a new influenza strain emerges with higher severity. The model, trained on previous seasons’ severity patterns, initially underestimates hospitalization demand. Human validation catches this discrepancy within 48 hours, prompting manual adjustments and communication to decision-makers about model limitations.

*Mitigation*: The incident illustrates the importance of human oversight, ensemble modeling (the team supplements the AI forecast with expert judgment and alternative models), and clear uncertainty communication (decision-makers understand that forecasts carry substantial uncertainty during novel epidemiological situations).

## Evaluation and Continuous Learning

Systematic evaluation is essential for responsible AI deployment in public health emergencies. Agencies should conduct pre-deployment testing through prospective validation using historical emergency data, simulation exercises, and tabletop drills to assess accuracy, identify failure modes, and evaluate integration with decision workflows before operational use. Performance benchmarking should establish baseline accuracy metrics, track performance over time, and, where possible compare AI-supported versus non-AI-supported decision outcomes to assess actual impact on decision quality. Real-time monitoring requires dashboards that track model performance during deployment with automated alerts for degradation, supplemented by regular expert review to identify issues not captured by automated metrics. Agencies must establish adverse event reporting mechanisms similar to health care systems, with clear protocols for investigating suspected AI-related errors or harms and implementing corrective actions. Post-emergency after-action reviews should systematically assess whether AI tools were useful, what worked well, what failed, how tools should be modified, and whether unintended consequences or equity impacts occurred. Finally, agencies should define clear decommissioning criteria and transition plans for retiring models when they exhibit poor performance, conditions change, or better alternatives become available.

## Policy Implications of AI in Public Health Decision Making

State-level legislative activity around governmental AI use has accelerated rapidly, with more than 150 bills introduced in 2024 and at least 30 states issuing formal guidance on AI use by state agencies. For example, Connecticut has implemented the AI Responsible Use Framework (Policy AI-01), which promotes ethical AI use, fairness, privacy, and transparency across all state agencies. Maryland enacted the Artificial Intelligence Governance Act of 2024, requiring each unit of state government to conduct inventories and assessments of systems that employ high-risk AI, and providing for the Department of Information Technology to develop policies and procedures concerning the development, procurement, deployment, use, and assessment of such systems [38]. Vermont established the Division of Artificial Intelligence within the Agency of Digital Services to review all aspects of AI systems developed, employed, or procured in state government [38].

At the federal level, several policies regulate the use of AI in governmental decision-making. The AI in Government Act of 2020 facilitates the adoption of AI technologies in the federal government, aiming to improve cohesion and competency in the adoption and use of AI within federal agencies. Executive Order 13960, titled “Promoting the Use of Trustworthy Artificial Intelligence in the Federal Government,” establishes the policy of the United States to promote the innovation and use of AI to improve government operations and services in a manner that fosters public trust and confidence while protecting privacy, civil rights, civil liberties, and American values. Additionally, the Office of Management and Budget (OMB) issued Memorandum M-25‐21, “Accelerating Federal Use of AI through Innovation, Governance, and Public Trust,” which provides guidance for federal agencies to enhance their AI governance and risk management practices [39-41]. More recently, Executive Order 14,319 (July 2025), titled “Preventing Woke AI in the Federal Government,” required federal agencies to procure only AI models that prioritize truth-seeking and ideological neutrality, restricting the incorporation of diversity, equity, and inclusion frameworks into AI outputs [42]. This policy places constraints on public health AI decision-making by limiting the use of equity-informed analytic approaches that are commonly used to identify health disparities and guide targeted interventions. In December 2025, Executive Order 14365, titled “Ensuring a National Policy Framework for Artificial Intelligence,” established a uniform national AI framework intended to preempt state regulations deemed burdensome to innovation. By authorizing federal challenges to inconsistent state laws and allowing the withholding of federal funding from non-compliant states, the order reshapes public health AI governance by centralizing oversight, potentially reducing states’ ability to tailor AI safeguards, transparency standards, and equity protections to local public health needs [43].

[38]To strengthen AI regulations in government, several policies should be implemented. Governments should establish ethical AI frameworks that operationalize the fairness and bias mitigation principles discussed earlier, with continuous assessments to identify biases, ensure privacy protection, and evaluate AI system reliability. More states should implement policies like Connecticut, Maryland, and Vermont to regulate AI use within state agencies to ensure the ethical use of AI systems in government operations [38]. Additionally, mandatory impact assessments should be conducted before deploying AI systems, ensuring that risks, including bias and discrimination, are mitigated and disclosed publicly. Governments should also require AI systems to be explainable, allowing the public to understand the decision-making logic behind AI outcomes. For instance, New York has required state agencies to publicly disclose detailed information about their automated decision systems [44]. Public oversight should be ensured through independent governance structures, such as Chief AI Officers, who would be responsible for ensuring AI deployment aligns with ethical standards and transparency.

Building public trust in AI use requires active engagement with stakeholders, including community leaders, ethicists, and public health experts, to ensure AI applications meet the needs and values of the community. Governments must prioritize clear and accessible communication about how AI is used in decision-making, including detailed reports on AI system performance, challenges, and successes. Regular audits should be conducted to address potential biases, ensuring AI systems are fair and inclusive. Furthermore, ethical AI governance structures should be implemented, including ethics boards and external reviews, to monitor and guide responsible AI use. Creating avenues for public feedback, such as town halls or online platforms, will allow communities to voice concerns and provide input on improving AI systems. Strong privacy protections should be enacted to safeguard citizens’ data, ensuring AI systems are compliant with privacy standards. Lastly, ongoing public education and training on AI literacy will help demystify AI and foster confidence in its use, ultimately increasing public trust in governmental AI decision-making.

However, even with emerging governance frameworks, empirical evidence suggests that formal governance structures do not automatically translate into improved outcomes [45-48]. Cross-country studies find that standard governance indicators (government effectiveness, rule of law, transparency) show weak or inconsistent relationships with population health outcomes once other factors are controlled. Similarly, AI governance arrangements (such as ethical guidelines, transparency principles, and oversight bodies) do not necessarily translate into effective implementation at the operational level. This disconnect occurs because high-level institutional indicators may fail to capture sector-specific capacities essential for public health emergency response: data quality, frontline health system integration, decision-maker competence, organizational culture, and implementation fidelity. Successful AI governance requires attention to these operational realities, not just policy documents.

## The AI Decision Support Lifecycle for Public Health Emergencies

**Figure 1.**
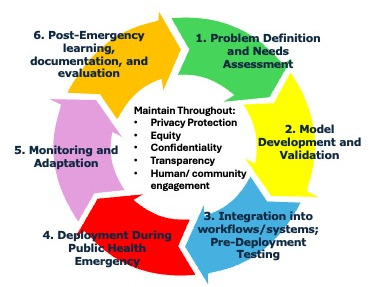
AI decision supported lifecycle in public health emergencies.

Drawing on the technical, ethical, organizational, and policy considerations discussed above, we propose an AI Decision Support Lifecycle framework specifically designed for governmental public health emergency response (Figure 1).

*Phase 1* consists of defining the problem at hand and conducting a needs assessment. Stakeholders must clarify the decisions that will be made, the information that is required to help decision makers, the end users of AI outputs, and the consequences of wrong decisions. This phase involves engaging elected officials, public health leaders, emergency managers, and community representatives to ensure AI development targets genuine decision needs.

*Phase 2* consists of equity-driven, technically robust model development and validation. Key details and steps include using representative data, evaluating performance across demographic and geographic subgroups, and documenting model limitations and uncertainty. Additional safeguards include independent expert review prior to operational deployment.

*Phase 3* includes integrating AI models into workflows and pre-deployment testing. AI tools must be integrated into existing workflows and information systems. Steps include but are not limited to developing user interfaces compatible with incident command structures, training end users on interpretation and appropriate use, and establishing clear protocols for human validation and override of AI recommendations.

In *Phase 4***,** a model is ready for deployment during an emergency. During an emergency, AI tools provide decision support while human officials retain ultimate authority. When AI is being deployed during an emergency, model performance must be monitored in real time, validating outputs using expert evaluation before high-stakes decisions, and clear communication of uncertainty to decision makers.

Monitoring and adaptation comprise *Phase 5*, which includes tracking accuracy metrics, comparing predictions to observed outcomes, and adjusting models as new data becomes available.

Finally, in *Phase 6*, models are evaluated after emergency deployment, and lessons learned are gleaned. Evaluation includes understanding the model’s usefulness for decision making, identifying what worked well and what didn’t as well as any unintended consequences, and understanding how the tool may be used or modified for future emergencies.

A number of critical principles apply throughout the phases of the AI Decision Support Lifecycle for Public Health Emergencies. First, it is vital that there are robust privacy and confidentiality protections in place and that there is limited data use for limited and specific purposes. Next, equity principles should be positioned as a key priority in model development and implementation. Similarly, there must be clear documentation for model development and use, explainability for model outputs, and public accountability for the use of AI in public health decision-making. Finally, human authority must remain the centerpiece of public health emergency decision-making. AI can only serve as decision support, never an autonomous decision-maker.

Table 1 organizes and synthesizes technical, ethical, organizational, and policy challenges that occur throughout the lifecycle, with specific actionable recommendations for model developers, health agencies, and elected officials.

**Table 1.** Key challenges and stakeholder actions for AI integration in public health emergency decision-making.

Challenge area	Model developers	Health agencies & officials	Policymakers & elected officials
Technical challenges			
Model Development (underfitting, overfitting, data quality)	Ensure models are sensitive to data quality and representativeness; avoid over-specialization to training data	Work with modelers to validate models before deployment; establish validation processes for AI outputs	Understand model limitations; ensure AI enhances rather than supplants judgment
Ethical challenges			
Bias & Fairness	Curate representative datasets; assess for performance disparities across subgroups; document model development transparently	Continuously evaluate models for bias; implement continuous monitoring and regular auditing of AI systems	Ensure decisions account for diverse communities; conduct regular audits for fairness and inclusiveness
Privacy & Data Security	Design models with privacy protections; maintain confidentiality of training and testing data	Implement robust data management infrastructure and cybersecurity protections	Enforce transparent risk management and privacy protection policies; ensure civil rights protections
Organizational challenges			
Workforce Development & AI Literacy	Design user-friendly interfaces; provide training resources for end users	Implement foundational AI literacy programs; provide expert workshops using scenario-based training	Understand AI’s potential and limitations; receive training to interpret and implement AI outputs
Workflow Integration & Implementation	Include human validation functions, feedback loops, and ensemble functions combining model predictions with expert systems	Integrate AI outputs into crisis-management dashboards; ensure seamless alignment with existing processes	Establish governance frameworks to reduce hesitation in supporting AI-derived recommendations
Resource Allocation & Infrastructure	Plan for ongoing maintenance and model updates beyond initial development	Invest in data management infrastructure, cybersecurity, and dedicated personnel	Allocate resources beyond software licensing and hardware acquisition
Policy & governance challenges			
Governance Frameworks & Standards	Follow ethical AI principles prioritizing fairness, transparency, and accountability	Develop adaptive governance structures; implement continuous risk assessments	Establish ethical AI frameworks at federal and state levels; implement mandatory impact assessments
Public Trust & Stakeholder Engagement	Design transparent systems that enable public understanding of AI decision-making processes	Engage with community leaders, ethicists, and public health experts; provide detailed performance reports	Ensure AI applications meet community needs and values; prioritize clear communication about AI use

## Conclusion

Artificial intelligence holds substantial promise for supporting governmental public health emergency response, but responsible implementation requires adherence to core ethical principles rather than simply deploying technology. First, AI must function strictly as decision-support tools while preserving human authority. Elected officials and public health leaders retain ultimate accountability for decisions, with clear protocols for validating and overriding AI recommendations. Second, equity must be designed into AI systems from inception, not treated as an afterthought, through representative data, subgroup performance evaluation, community advisory boards, and transparent equity impact reporting. Third, successful AI deployment demands comprehensive organizational readiness, including workforce capacity (data scientists working collaboratively with epidemiologists and communication specialists), robust data infrastructure, governance structures, and sustained funding beyond initial acquisition costs.

Fourth, transparency and accountability mechanisms are non-negotiable, requiring public disclosure of when AI informs decisions, comprehensive model documentation, explainable outputs, and clear processes for challenging AI-informed decisions. Finally, continuous monitoring, evaluation, and adaptation throughout the AI lifecycle ensures systems remain reliable as conditions change, with pre-deployment testing, real-time performance monitoring, adverse event reporting, and willingness to decommission underperforming models. Achieving AI’s potential requires genuine collaboration between technical developers, public health agencies, and elected officials, with each stakeholder group taking specific, complementary actions to ensure models meet legal and ethical standards while meaningfully supporting evidence-based decision-making that reduces rather than exacerbates health inequities.

## Abbreviations

AI: artificial intelligence
ICT: information and communication technology
ML: machine learning
OMB: Office of Management and Budget

## References

[1] (2025). US centers for disease control and prevention. Public Health Emergency Preparedness and Response Capabilities.

[2] Nelson C, Lurie N, Wasserman J, Zakowski S (2007). Conceptualizing and defining public health emergency preparedness. Am J Public Health.

[3] Hodge JG, Dunning LT, Piatt JL (2023). State public health emergency powers in response to COVID-19. Am J Public Health.

[4] Orenstein DG (2013). When law is not law: setting aside legal provisions during declared emergencies. J Law Med Ethics.

[5] Hodge JG, Anderson ED (2009). Principles and practice of legal triage during public health emergencies [Abstract].

[6] Sunshine G, Barrera N, Corcoran AJ, Penn M (2019). Emergency declarations for public health issues: expanding our definition of emergency. J Law Med Ethics.

[7] Higgins G, Freedman J (2013). Improving decision making in crisis. JBCEP.

[8] Agarwal R, Gao G (Gordon, DesRoches C, Jha AK (2010). **Research Commentary** —The digital transformation of healthcare: current status and the road ahead. Information Systems Research.

[9] Cline GB, Luiz JM (2013). Information technology systems in public sector health facilities in developing countries: the case of South Africa. BMC Med Inform Decis Mak.

[10] Sheikh A, Sood HS, Bates DW (2015). Leveraging health information technology to achieve the “triple aim” of healthcare reform. J Am Med Inform Assoc.

[11] Fichman RG, Kohli R, Krishnan R (2011). **Editorial Overview** —The role of information systems in healthcare: current research and future trends. Information Systems Research.

[12] Scott RE, Mars M (2013). Principles and framework for eHealth strategy development. J Med Internet Res.

[13] OECD, UNESCO, G7 Italia (2024). G7 toolkit for artificial intelligence in the public sector. https://www.oecd.org/en/publications/g7-toolkit-for-artificial-intelligence-in-the-public-sector_421c1244-en.html.

[14] Chen Z, Zhang JM, Sarro F, Harman M (2023). A comprehensive empirical study of bias mitigation methods for machine learning classifiers. ACM Transactions on Software Engineering and Methodology.

[15] Benjamin R (2019). Assessing risk, automating racism. Science.

[16] Mhasawade V, Zhao Y, Chunara R (2021). Machine learning and algorithmic fairness in public and population health. Nat Mach Intell.

[17] McCradden MD, Joshi S, Anderson JA, Mazwi M, Goldenberg A, Zlotnik Shaul R (2020). Patient safety and quality improvement: Ethical principles for a regulatory approach to bias in healthcare machine learning. J Am Med Inform Assoc.

[18] Obermeyer Z, Powers B, Vogeli C, Mullainathan S (2019). Dissecting racial bias in an algorithm used to manage the health of populations.

[19] Young JC, Conover MM, Funk MJ (2018). Measurement error and misclassification in electronic medical records: methods to mitigate bias. Curr Epidemiol Rep.

[20] Campbell EA, Bose S, Masino AJ (2024). Conceptualizing bias in EHR data: A case study in performance disparities by demographic subgroups for a pediatric obesity incidence classifier. PLOS Digit Health.

[21] Andaur Navarro CL, Damen JAA, Takada T (2021). Risk of bias in studies on prediction models developed using supervised machine learning techniques: systematic review. BMJ.

[22] Pathak YV, Saikia S, Pathak S, Patel JK, Prajapati JB (2023). Ethical Issues in AI for Bioinformatics and Chemoinformatics.

[23] London AJ (2019). Artificial intelligence and black-box medical decisions: accuracy versus explainability. Hastings Cent Rep.

[24] Gerke S, Minssen T, Cohen G (2020). Ethical and legal challenges of artificial intelligence-driven healthcare. Artif Intell Healthc.

[25] Martin KD, Zimmermann J (2024). Artificial intelligence and its implications for data privacy. Curr Opin Psychol.

[26] Oseni A, Moustafa N, Janicke H, Liu P, Tari Z, Vasilakos A (2021). Security and privacy for artificial intelligence: opportunities and challenges. arXiv.

[27] Bak M, Madai VI, Fritzsche MC, Mayrhofer MT, McLennan S (2022). You can’t have ai both ways: balancing health data privacy and access fairly. Front Genet.

[28] Yadav N, Pandey S, Gupta A, Dudani P, Gupta S, Rangarajan K (2023). Data privacy in healthcare: in the era of artificial intelligence. Indian Dermatol Online J.

[29] Curzon J, Kosa TA, Akalu R, El-Khatib K (2021). Privacy and artificial intelligence. IEEE Trans Artif Intell.

[30] Andreotta AJ, Kirkham N, Rizzi M (2022). AI, big data, and the future of consent. AI Soc.

[31] Adapa VR (2024). Navigating the privacy paradox: balancing ai advancement and data protection in the digital age. Int J Sci Res Comput Sci Eng Inf Technol.

[32] Stanford V, Gresh L, Toledo J, Méndez J, Aldighieri S, Reveiz L (2022). Evidence in decision-making in the context of COVID-19 in Latin America. Lancet Reg Health Am.

[33] Hu Y, Jacob J, Parker GJM, Hawkes DJ, Hurst JR, Stoyanov D (2020). The challenges of deploying artificial intelligence models in a rapidly evolving pandemic. Nat Mach Intell.

[34] Medaglia R, Zheng L (2017). Mapping government social media research and moving it forward: a framework and a research agenda. Gov Inf Q.

[35] Ding X, Shang B, Xie C, Xin J, Yu F (2025). Artificial intelligence in the COVID-19 pandemic: balancing benefits and ethical challenges in China’s response. Humanit Soc Sci Commun.

[36] McAndrew T, Gibson GC, Braun D, Srivastava A, Brown K (2024). Chimeric Forecasting: an experiment to leverage human judgment to improve forecasts of infectious disease using simulated surveillance data. Epidemics.

[37] Roy K, Zhang Q, Gaur M, Sheth A (2021). Knowledge infused policy gradients for adaptive pandemic control. arXiv.

[38] Hooshidary S, Canada C, Clark W Artificial intelligence in government: the federal and state landscape. NCSL.

[39] Campbell EA, Holl F, Marwah HK, Fraser HS, Craig SS (2025). The impact of climate change on vulnerable populations in pediatrics: opportunities for AI, digital health, and beyond—a scoping review and selected case studies. Pediatr Res.

[40] (2025). United States White House. Removing barriers to American leadership in artificial intelligence.

[41] United States White House. White House releases new policies on federal agency AI use and procurement.

[42] (2025). United States White House. Preventing Woke AI in the Federal Government.

[43] (2025). United States White House. Ensuring a National Policy Framework for Artificial Intelligence.

[44] (2025). Erence of state legislatures. Artificial Intelligence 2025 Legislation.

[45] Deaton A (2013). The Great Escape: Health, wealth, and the origins of inequality.

[46] Filmer D, Pritchett L (1999). The impact of public spending on health: does money matter?. Soc Sci Med.

[47] Wagstaff A (2002). Poverty and health sector inequalities. Bull World Health Organ.

[48] Holmberg S, Rothstein B (2011). Dying of corruption. Health Econ Policy Law.

